# Phylogenetic Relationships of the Triassic *Archaeosemionotus* Deecke (Halecomorphi, Ionoscopiformes) from the ‘Perledo Fauna’

**DOI:** 10.1371/journal.pone.0108665

**Published:** 2014-10-08

**Authors:** Adriana López-Arbarello, Rudolf Stockar, Toni Bürgin

**Affiliations:** 1 SNSB- Bavarian State Collection for Palaeontology and Geology, and GeoBio-Center Ludwig Maximilian University, Munich, Germany; 2 Museo Cantonale di Storia Naturale, Lugano, Switzerland; 3 Naturmuseum, Gallen, Switzerland; Universität Göttingen, Germany

## Abstract

The lagerstätten in the Monte San Giorgio have provided excellent fossils representing one of the most important windows to the marine life during the Triassic. Among these fossils, fishes are abundant and extraordinarily well preserved. Most of these fishes represent extinct lineages and were difficult to understand and classify during the early years after discovery. These difficulties usually led to a mixture of species under the same taxonomic name. This is the case of fishes referred to the genus *Archaeosemionotus*. The name bearing type of *A. connectens*, the type species of this genus, represents a basal halecomorph, but most other fishes referred to this genus represent basal ginglymodians. Therefore, we conducted this study to clarify the taxonomic status and phylogenetic relationships of *A. connectens*, which is a member of the family Furidae (Halecomorphi, Ionoscopiformes) representing the second cladistically supported evidence of ionoscopiforms in the Triassic and it is thus one of the two oldest reliable records of this group. Ionoscopiforms have a long stratigraphic range, though their fossil record is rather patchy. In our analysis, the sister taxon of *Archaeosemionotus* is *Robustichthys* from the Anisian of China, and they together form a clade with *Furo*, which is known from several localities ranging from the Early to the Late Jurassic. Other ionoscopiforms are so far known from the Kimmeridgian to the Albian and it is thus evident that recent efforts have concentrated on the later history of the group (Late Jurassic to Cretaceous). The phylogenetic relationships obtained for the Ionoscopiformes do not show a clear palaeobiogeographic pattern, but give important new insights into the origin, divergence date and early history of this clade.

## Introduction

The so-called ‘Perledo fauna’ was collected before the middle of the XIX century from small quarries opened in the area of Perledo, Varenna (Northern Italy), which were intended for the production of slabs and ornament stones. Recent works [1], [2], [3] stressed that these fossils actually represent at least three different assemblages within the Perledo-Varenna Formation, which are usually mixed up in historical collections. The first two fossil assemblages belong to the Varenna Limestone (the lower member of the Formation consisting of well-bedded limestones) and possibly correspond to the assemblages of the Besano Formation (earliest Ladinian [4]) and the Lower Meride Limestone in the area of the Monte San Giorgio (early Ladinian [5]). The younger assemblage belongs to the uppermost part of the Perledo-Varenna Formation, the up to 100 m thick Perledo Member, characterized by dark, finely laminated limestone with thin-bedded shale intercalations. The Perledo Member yielded most of the classical ‘Perledo fauna’ and it is dated as late Ladinian [6] and possibly correlates with the uppermost Meride Limestone (“Kalkschieferzone” [1], [7]).

The fishes of the ‘Perledo fauna’ were first published by Balsamo-Crivelli [8], who studied two specimens referring one of them tentatively to the genus *Semionotus* and the other as a new species *Lepidotus trotti* (currently *Furo trotti* after [3]). However, the first thorough study of these fishes was done by Bellotti [9], who described 14 new species of actinopterygians grouped in four genera (Table 1 in Table S1). In 1873 Bellotti completed a catalogue of the fossil fishes of the Museo di Storia Naturale di Milano, but this work was only published by Pinna in 1991 [10]. In this work, Bellotti added two new nominal species (Table 1 in Table S1). These names take authority in Bassani [11] because this is the first publication in which the names were actually published. The first revision of the fishes of the ‘Perledo Fauna’ was carried out by Deecke [12], but he did not have access to the specimens studied by Bellotti [9] and based his studies on the material available to him in the collections of the Senckenbergischen Museum (currently Forschungsinstitut Senckenberg (SMF), Frankfurt am Main, Germany) and the Strassburger Universitätssammlung. In this revision, which is part of a more general study on Triassic fishes, Deecke created three new genera, *Archaeosemionotus*, *Allolepidotus* and *Prohalecites*, which are currently valid, and four new species, three of which are also still valid (Table 2 in Table S1). A more complete revision of the fishes from Perledo was done by De Alessandri [13] who further added four new nominal species to the already quite diverse fauna of actinopterygians (Table 3 in Table S1).

The taxonomic history of most of the actinopterygians of the ‘Perledo fauna’ is complicated and difficult to trace back. The numerous nominal species have been mixed up by different authors based on different collections [3] and since most of the type material has been lost, the taxonomic status of many of those nominal species is dubious (Table S1). The present contribution aimes to clarify the taxonomic status and phylogenetic relationships of *Archaeosemionotus connectens* Deecke [12], which has one of the most conflicting taxonomic histories among the fishes from Perledo. The present systematic revision is based on the poorly preserved, though still diagnosable holotype, which is housed at the Senckenberg Research Institute and Natural History Museum (SMF) and an incomplete, but very well preserved specimen of this species in the Paleontological Institute and Museum at the University of Zürich (PIMUZ).

## Materials and Methods

The specimens were studied under a Leica M80 binocular microscope. Drawings were made with a Wild 308700 camera lucida and later digitized. Photographs were made with a Nikon D5100 camera with an AF-S VR Micro-Nikkor lens. Measurements were taken with a vernier calliper. Skull bones are named according to the use of most authors in actinopterygians.

### Anatomical abbreviations

**ag**, angular; **b.fu**, basal fulcra; **ch**, ceratohyal; **cl**, cleithrum; **cor**, coronoids; **d**, dentary; **d.c.fu**, dorsal caudal fulcra; **dpt**, dermopterotic; **dsp**, dermosphenotic; **br**, branchiostegal rays; **fr.fu**, fringing fulcra; **g**, gular plate; **io**, infraorbitals; **iop**, interoperculum; **mx**, maxilla; **op**, operculum; **pcl**, postcleithra; **pmx**, premaxillae; **pop**, preoperculum; **p.r**, principal ray; **q**, quadrate; **sag**, surangular; **sc**, scute; **scl**, supracleithrum; **smx**, supramaxilla; **so**, supraorbitals; **sop**, suboperculum; **s.r**, scale-like ray; **suo**, suborbitals.

### Cladistic analysis

To explore the phylogenetic relationships of *Archaeosemionotus connectens* we performed a cladistic analysis based on parsimony. For the analysis we assembled a data matrix of 57 characters and 21 taxa using Mesquite Version 2.75 [14]. This data matrix is a subsample of a larger matrix compiled by merging the data matrices for amiiforms of Grande and Bemis [15], the data matrix for ophiopsids of Alvarado-Ortega and Espinosa-Arrubarrena [16], and by adding *Archaeosemionotus connectens* and *Furo muensteri* [17] from the Late Jurassic of southern Germany, and the recently described *Robustichthys luopingensis* [18], from the Middle Triassic of southern China (Text S1). Character scores for the latter taxa are based on Lane and Ebert [19] and Xu et al. [18], respectively. The data matrix is available in Morphobank under Project 1105 [20].

Tree search was performed with PAUP* Version 4.0 beta version [21]. All characters were considered unordered and given equal weight. Most parsimonious trees were obtained through branch-and-bound search with furthest addition sequence. The distribution of characters and character changes have been analysed in PAUP* through accelerated and decelerated transformations (ACCTRAN and DELTRAN respectively; see list of synapomorphies in the Text S1) and with the “Trace Character” option in Mesquite. Branch support was evaluated through decay indexes for each node (Bremer support) and bootstrap and jackknife methods through branch-and-bound search with furthest addition sequence.

## Results

### Systematic Palaeontology

Neopterygii Regan 1923 [22]

Holostei Müller 1846 [23] (sensu Huxley [24])

Halecomorphi Cope 1887 [25] (sensu Grande and Bemis [15])

Ionoscopiformes Grande and Bemis 1998 [15]

Furidae Jordan 1923 [26]

***Archaeosemionotus*** Deecke 1889 [12]

**Type species**. *Archaeosemionotus connectens* [12]

**Diagnosis**. As for the type and only species.

***Archaeosemionotus connectens*** Deecke 1889 [12]

(Figs 1–5)

**Figure 1:**
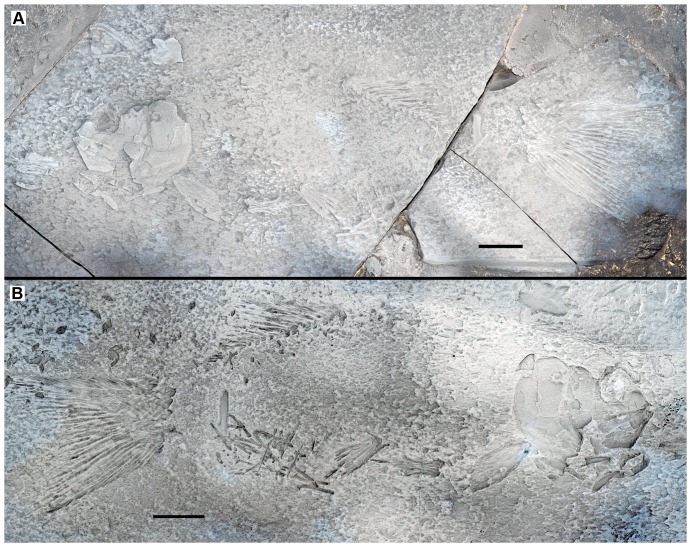
Holotype of *Archaeosemionotus connectens*. Specimen (SMF-P1238; c. 115 mm SL) preserved in left lateral view (**A**) with counter slab (**B**). Both slabs are dusted with ammonium chloride. Scale bars  = 1 cm. [planned for 2-column width].

**Figure 2:**
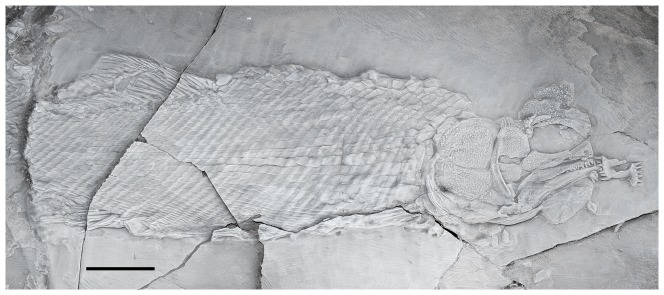
Specimen PIMUZ A/I 552 of *Archaeosemionotus connectens*. Specimen preserved in right lateral view and dusted with ammonium chloride. Scale bar  = 1 cm. [planned for 2-column width].

**Figure 3:**
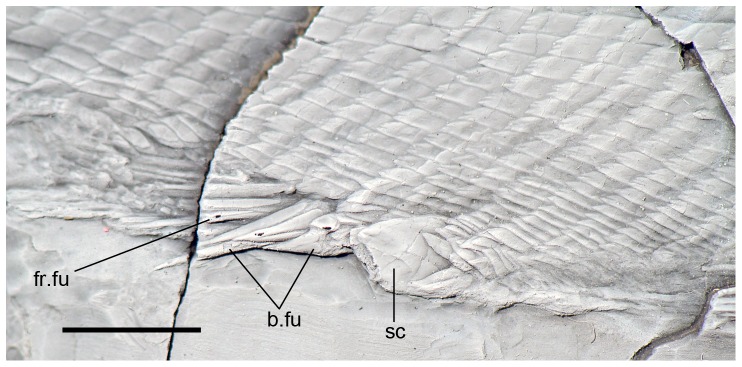
Anal scute and ventral scales in *Archaeosemionotus connectens*. Specimen PIMUZ A/I 552 dusted with ammonium chloride. Scale bar  = 1 cm. [planned for 1,5-column width].

**Figure 4:**
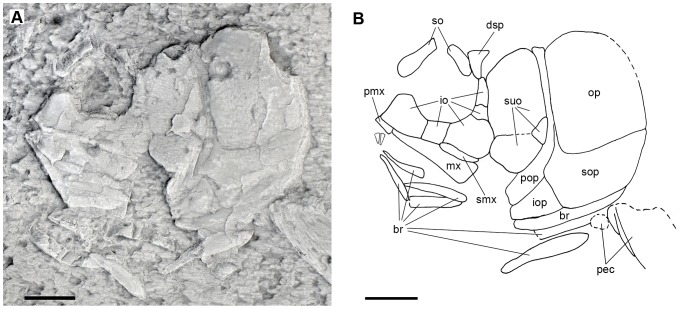
Skull of the holotype of *Archaeosemionotus connectens*. **A**, photograph of the specimen dusted with ammonium chloride; **B**, line drawing of the same specimen. Dotted lines indicate broken or reconstructed borders of bones. Scale bars  = 5 mm. [planned for 2-column width].

**Figure 5:**
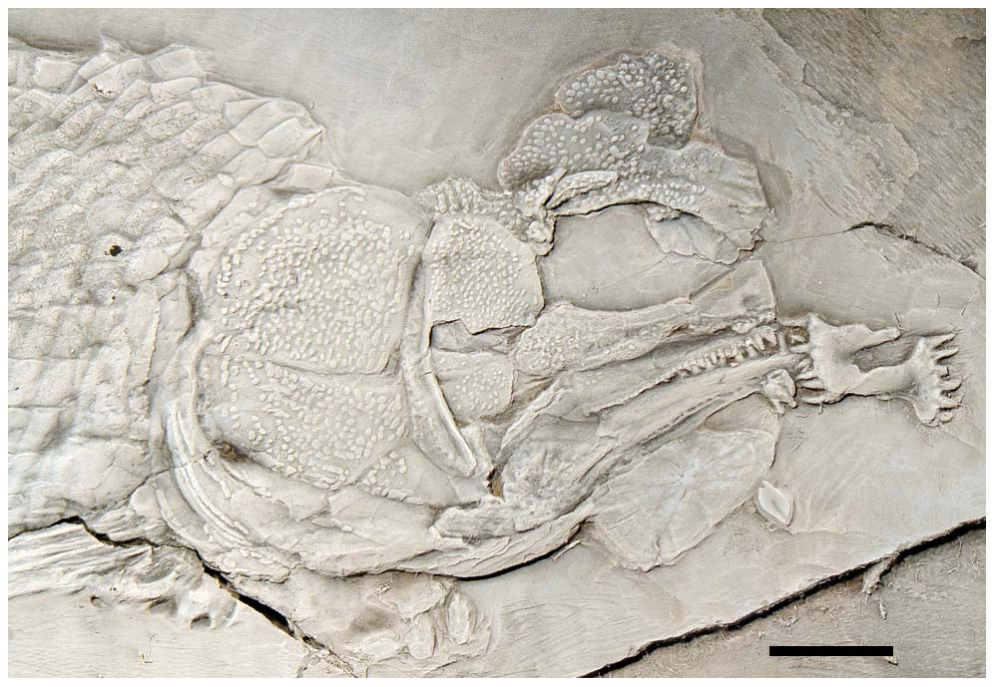
Photograph of the skull skull of PIMUZ A/I 552 of *Archaeosemionotus connectens*. Scale bar  = 5 mm. [planned for 1-column width].

*Archaeosemionotus connectens* Deecke [12]: p. 121, table 6, figure 3.

*Archaeosemionotus connectens* De Alessandri [13]: p. 71.

*Archaeosemionotus connectens* Bürgin [27]: p. 955.

*Archaeosemionotus connectens* Tintori and Lombardo [28]: p. 370.

**Holotype.** SMF-P1238a/b; very incomplete specimen preserved in left lateral view in part and counterpart (Fig. 1).

**Referred material.** PIMUZ A/I 552; incomplete, but very well preserved specimen from the Perledo Member of the Perledo-Varenna Formation. The fish is preserved in right lateral view; the caudal fin is missing (Fig. 2).

**Diagnosis.** Small fusiform neopterygian (c. 115 mm SL) characterized by the following combination of characters (an asterisk “*” indicate the features observed in the holotype and referred specimen; a double S “§” indicates features observed in the holotype only): skull bones densely ornamented with relatively large tubercles*; relatively large infraorbital bones 1–3, forming the ventral margin of the orbit*; the infraorbital placed at the posteroventral corner (io3) of the orbit is expanded posteriorly*; maxilla with straight posterior border at the level of the posterior border of the orbit*; two large, dorsal and ventral suborbitals, the dorsal being twice the size of the ventral and a third small suborbital placed between the two larger suborbitals, at their posterior margins*; large median gular plate with straight posterior border; large suboperculum, approximately half the size of the operculum*; fringing fulcra present on all fins*; scales rhomboid, with smooth surfaces and strongly serrated posterior border; ventrum covered with several rows of distinctly shallow scales; 9 inverted rows of scales in the body lobe of the tail^§^.

**Type locality.** The quarry was at the left margin of the Como Lake between Varenna and Regoledo (translated from [12]).

**Type horizon.** Black shales of Perledo (translated from [12]).

The precise stratigraphic position of the specimens belonging to the ‘Perledo fauna’ within the 500 m thick Perledo-Varenna Formation is usually unknown. However, being embedded in a thin, dark slab, the holotype of *Archaeosemionotus connectens* may be tentatively attributed to the uppermost part of the Perledo-Verenna Formation (Perledo Member, late Ladinian [6]), which possibly correlates with the uppermost Meride Limestone (Kalkschieferzone, late Ladinian [1], [5], [7]).

### Description

Although the holotype specimen is incompletely preserved (Fig. 1), as indicated in the diagnosis, important anatomical information is observable in the skull and the caudal fin. Except for the caudal fin features, all other diagnostic features are present and better preserved in the Zürich specimen PIMUZ A/I 552 (Fig. 2) and, thus, the referral of the two specimens to the same species is straightforward (see also morphometric measurements in Table 4 in Table S1). Except when a particular specimen is indicated, the following description includes features visible in both specimens.

The almost complete absence of scales on the body of the holotype led Deecke [12] to interpret that the scales on the body of *Archaeosemionotus connectens* were probably weakly ossified and, therefore, not preserved in this specimen; alternatively, the body was naked. According to our observations, almost all the scales on the body lobe of the tail are preserved in the holotype and even a few body scales are also preserved articulated at the base of the caudal fin (Fig. 1). These few body scales are not weakly ossified, as interpreted by Deecke [12]. Additionally, there are several imprints of disarticulated rhomboid scales in the counter slab, although they are very difficult to distinguish and mainly become visible after dusting the specimen with ammonium chloride (Fig. 1B). According to Hutchinson [29], caudal and body squamation develop independently in actinopterygians with rhomboid scales. The caudal squamation starting at the tip of the body lobe and proceeding towards the hinge line, while the body squamation starts anteriorly and proceeds backwards. Therefore, the presence of those few body scales at the base of the caudal fin thus indicates that the holotype was probably an adult or sub-adult, with complete caudal and body squamation. Thus, the few body scales preserved at the base of the caudal fin indicate that the lack of scales on the body of the holotype do not represent a real absence, but rather incomplete preservation as already indicated by De Alessandri [13]. Furthermore, well-ossified rhomboid scales cover the whole body of the specimen PIMUZ A/I 552 (Fig. 2) showing that the squamation was complete in *Archaeosemionotus connectens*. There are 40 vertical rows of rhomboid scales in PIMUZ A/I 552, but the total number was certainly higher because the posterior portion of the caudal peduncle is missing in this specimen. The longitudinal row of scales carrying the lateral line is at the middle of the flank, but the number of longitudinal rows of scales above and below the lateral line is variable, ranging from about five rows immediately behind the skull to 13 at the origin of the dorsal fin and 11 at the end of the dorsal fin, and about 10 below the lateral line immediately behind the pectoral girdle to 20 at the pre-anal scute, and about eight at the end of the dorsal fin (uncertainties are due to poor preservation). Although the scales are all rectangular, their shape is very variable from slightly deeper than long in the most anterior portion of the flank (first 9 vertical rows) to longer than deep in the most part of the body, but specially very shallow in the ventrum and very particularly immediately anterior to the large scute that most probably covered the vent right before the origin of the anal fin (Fig. 3).

The body is long, shallow and fusiform. Several articulated skull bones including the tip of the snout and opercular series, as well as the body lobe, the caudal, dorsal and pelvic fins are preserved in situ as natural moulds in the counter slab of the holotype (SMF-P1238b; Fig. 1B). Therefore, body proportions are based on this specimen (see raw measurements taken on both specimens in Table 4 in Table S1). The standard length of the holotype is 107 mm (SL: measured from the tip of the snout to the base of the caudal fin at the hinge line). The head is relatively short, representing 28% of the SL and the eyes were relatively large, the length of the orbit being 26% of the length of the head. The dorsal fin originates at 60% of the SL. Based on this proportions and measurements taken on PIMUZ A/I 552, which preserves in situ remains of the anal and pelvic fins, we estimated that the pelvic fins of *A*. *connectens* insert approximately in the middle of the body at 55% of the SL, slightly anterior to the dorsal fin, and the anal fin originates at 75% of the SL.

**Figure 6:**
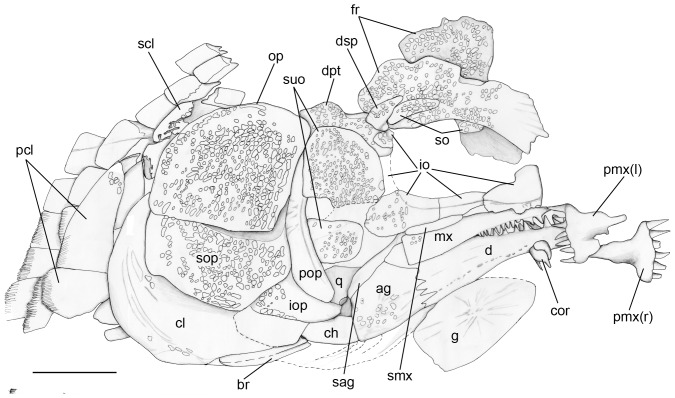
Line drawing of the skull of PIMUZ A/I 552 of *Archaeosemionotus connectens*. Dotted lines indicate broken or reconstructed borders of bones. Scale bar  = 5 mm. [planned for 2-column width].

Only several bones of the cheek and opercular apparatus are clearly distinguishable in the skull of the holotype (Fig. 4), but most of the skull is well preserved in PIMUZ A/I 552 (Figs. 5–6). The frontals, dermopterotic, circumborbital, suborbital and opercular bones are densely ornamented with relatively large tubercles covered with ganoine. Only the two frontals and the right dermopterotic remain of the skull roof in PIMUZ A/I 552. The frontals are large, asymmetric and relatively broad, widest posteriorly. They make a gently interorbital constriction. The anterior and posterior widths are about 30% and 40% of the length, respectively, in the right frontal, and about 35% and 63% in the left frontal. The dermopterotic is long and very narrow, the medial margin is excavated in the middle and the lack of ornamentation on the posterolateral margin suggests that it was probably overlapped by an extrascapular bone. The shape of the lateral margin of the frontals, which gently curves medially in the posterior portion, suggests that the anterior portion of the dermopterotic extended lateral to the frontal and articulated with the dermosphenotic. If so, although the parietals are not preserved, their length must have been shorter than the length of the dermopterotic. However, these features are uncertain and must be checked in articulated specimens, which are unfortunately not known yet.

The orbit is almost perfectly round and it was probably completely closed anteriorly, with the anterior supraorbital contacting the first infraorbital (SMF-P1238a/b; the orbit is deformed in PIMUZ A/I 552). The supraorbital bones are imperfectly preserved, but they were probably two. A relatively large and elongate anterior supraorbital bone is preserved at the anterodorsal rim of the orbit in SMF-P1238a/b (Fig. 4) and an elongate posterior supraorbital is preserved in PIMUZ A/I 552 (Fig. 5–6). In this later specimen, a fragment of an ornamented bone lateral to the right frontal at the interorbital constriction most probably represents the anterior supraorbital. There are five infraorbital bones. The infraorbitals 1–3 are very large, occupying the whole ventral margin of the orbit. The first infraorbital is triangular, highest anteriorly, with the anterior and ventral borders forming an almost right angle and the sensory canal is contained in a groove close to the ventral margin of the bone. The second infraorbital is long and shallow and the third infraorbital forms the posteroventral corner of the orbit and is expanded posteriorly. The second and third infraorbitals describe together an elongate posteroventrally smoothly lobulate shape. The infraorbital sensory canal is contained in a groove close to the dorsal borders of these two bones. The posterior margin of the orbit was formed by two small infraorbitals, which are indicated as impressions in SMF-P1238b (Fig. 4). In PIMUZ A/I 552 the fifth infraorbital is preserved ventral to the dermosphenotic, overlying the anterior portion of the dermopterotic, which is anteroventrally displaced, and there is an impression left by the fourth infraorbital, which was very narrow, deeper than long (Fig. 5–6). The dermosphenotic formed the posterodorsal corner of the orbit and is subtriangular in shape.

Two large suborbital bones occupy the area between the infraorbital bones and the preoperculum; the dorsal element is approximately two times larger than the ventral. A third and much smaller suborbital places between these two large suborbitals, but only at their posterior margins contacting the preoperculum (Figs. 4–6). The three suborbitals buttress the thickened anterior margin of the preoperculum. The preoperculum has a smoothly crescent shape and its ventral portion does not reach the level of the posterior border of the orbit. The operculum is broad, its maximal height is only 1.25 of its maximal length, and has gently rounded borders. The suboperculum is large, being about half the depth and as long as the operculum. The suboperculum is dorsally concave and ventrally convex, acuminating posteriorly, and has a well developed ascending process, which is partially hidden by the operculum. The interoperculum is small and approximately triangular, its length is 1.4 times its maximal, posterior depth; the anterior border is about one third of that depth. A large branchiostegal ray remains in position ventral to and partially overlapped by the inter- and suboperculum in the holotype (Fig. 4). Additionally, several small branchiostegals are preserved disarticulated and displaced in this specimen.

The two premaxillae and the right maxilla and supramaxilla are well preserved in PIMUZ A/I 552 (Figs. 5–6). Each premaxilla has a high nasal process, but the two nasal processes do not contact with each other and they are not perforated as in other halecomorphs or in ginglymodians. There is a single row of six large conical teeth on each premaxilla. The maxilla is shallow and triangular, with a straight posterior border at the level of the posterior margin of the orbit. There is a short articular process oriented anteromedially and a few pores in the anterior portion of the bone indicate the possible presence of a maxillary sensory canal, but better preserved material is necessary to confirm this feature. Only ten conical teeth are preserved in the anterior half of the maxilla, but several pits on the ventral border of the bone indicate that the tooth row reached the posterior border of the maxilla. The supramaxilla is long and shallow, oval in shape, resting on and extending along the posterior c. 40% of the dorsal border of the maxilla.

The dentary, angular and surangular form the lateral surface of the lower jaw (PIMUZ A/I 552). The coronoid process is gently rounded and its height is 38% of the lower jaw length. The posterior border of the dentary follows a zigzag line at the level of the posterior border of the maxilla. The dentary symphysis is shallow, about 38% of the height of the coronoid process and 15% of the lower jaw length. Nine strong conical teeth form a single row along the dorsal margin of the dentary. The posterodorsal portion of the dentary is laterally overlapped by the maxilla and, thus, the tooth row might have extended some further posteriorly. The dentary teeth are larger than the maxillary teeth, though they slightly decrease in size posteriorly, with the most anterior tooth being as large as the premaxillary teeth. Acrodine cups are not discernable in any of the premaxillary, maxillary or dentary teeth. The exposed portion of the surangular is long and shallow and the angular is large and massive. The facet for articulation with the quadrate is not visible and, thus, it was probably oriented medially. The quadrate is small and has a poorly defined condyle, which is oriented almost vertically.

As usual, the cleithrum is the largest bone of the pectoral girdle. It has a crescent shape in lateral view, extending from approximately a level a little above the ventral border of the operculum up to almost the anterior end of the interoperculum. The lateral wing of the cleithrum is broad and ornamented with elongated parallel ridges, which are aligned following the crescent shape of the bone. The cleithrum is expanded medially, but do not form a median wing as in ginglymodians. The supracleithrum is incompletely exposed, being partially hidden by the operculum. The exposed surface of the supracleithrum is ornamented with irregular ganoine patches. There are two large postcleithra and fragments of two possible additional postcleithra posteroventral to the cleithrum. The largest postcleithra is the most dorsal element in the series. This dorsal postcleithrum is subtriangular in shape, broadest ventrally and elongated dorsoventrally. The second postcleitrhum is about a third of the size of the dorsal postcleithrum and it is longitudinally elongated. The two following postcleithra are small, approximately as large as the flank scales. The pectoral fins cannot be described because they are too poorly preserved in both specimens. The pelvic girdles are not preserved in the holotype and they are completely covered by scales in PIMUZ A/I 552. The pelvic fins are only preserved as imprints in the holotype (SMF-P1238b). There are at least five fin rays and a series of fringing fulcra.

**Figure 7:**
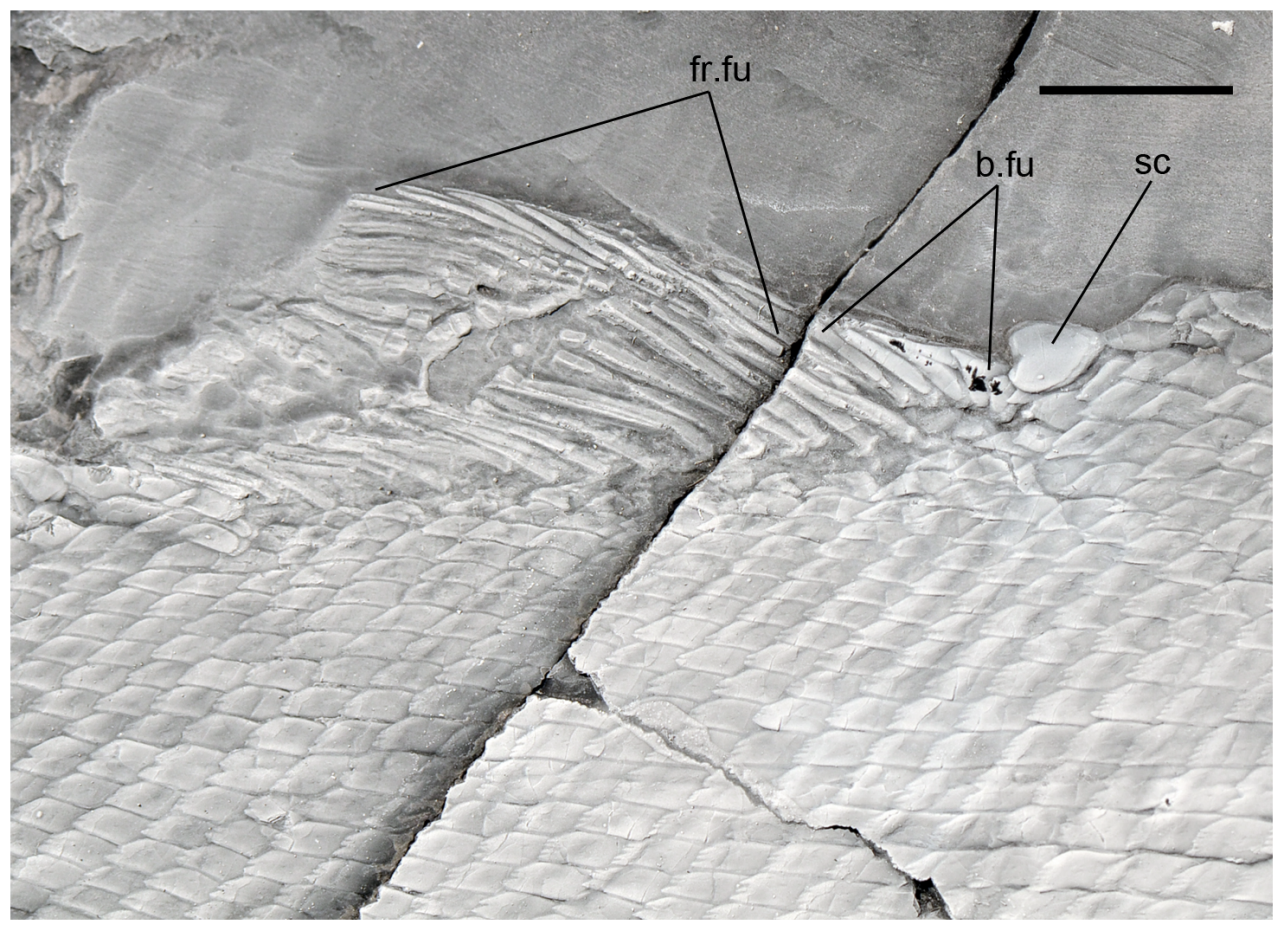
Dorsal fin of *Archaeosemionotus connectens*. Photograph of the specimen PIMUZ A/I 552 dusted with ammonium chloride; **B**, line drawing of the same specimen. Scale bar  = 5 mm. [planned for 1-column width].

The dorsal and anal fins are best preserved in PIMUZ A/I 552. In this specimen there is a heart-shaped predorsal scute, which is only a little larger than the normal flank scales (Fig. 7). The dorsal fin begins with three small, unpaired basal fulcra followed by one paired basal fulcrum and seven long and slender fringing fulcra. There are at least 16 dorsal fin rays. The anal fin is preceded by one large median pre-anal scute (Fig. 3). The anal fin starts with one unpaired basal fulcrum, which is followed by two slender paired basal fulcra and a series of slender fringing fulcra. The number of anal fin rays is unknown.

**Figure 8:**
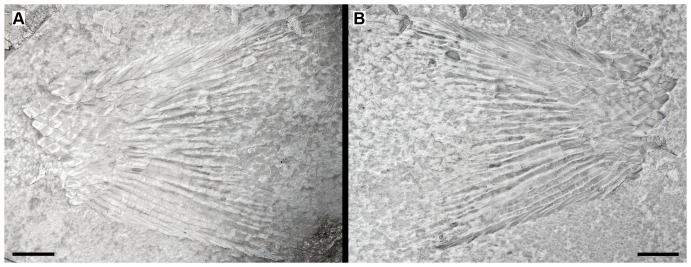
Caudal fin of *Archaeosemionotus connectens*. Photographs of the holotype specimen dusted with ammonium chloride **A**, slab (SMF-P1238a); **B**, counter slab (SMF-P1238b). Scale bars  = 5 mm. [planned for 2-column width].

**Figure 9:**
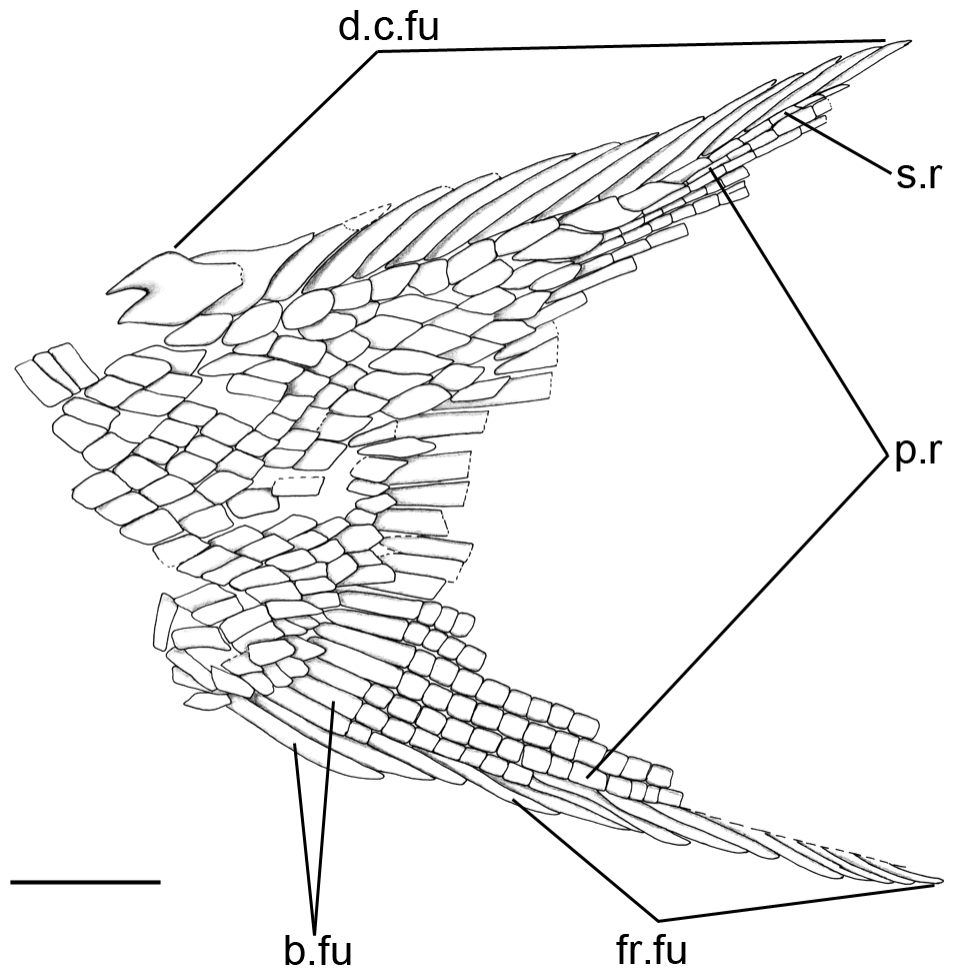
Line drawing of the caudal fin of *Archaeosemionotus connectens*. The drawing was made with camera lucida under the microscope on the slab of the holotype (SMF-P1238a) and it was later completed overlapping the first drawing on the reflection of the counter slab (SMF-P1238b). Scale bar  = 5 mm. [planned for 1-column width].

The caudal fin is abbreviated heterocercal and it is only preserved in the holotype (Figs. 8–9). The fin is deeply forked and made of 22 principal rays. The body lobe is formed by nine inverted rows of caudal scales and it is long, with a ray-like distal tip resembling the scale-like ray of giglymodians [30], [31]. The longest among these rows includes c. 12 scales and it is flanked by a marginal row of c. 7 scales, which does not reach the tip of the body lobe. The size and shape of the scales of the body lobe is similar to that of the body scales on the caudal peduncle. The dorsal margin of the fin is garnished with a series of dorsal caudal fulcra (14 are preserved, but the more distal elements are missing). The 10 anterior dorsal caudal fulcra are inserted on the body lobe and the following elements of this series are fringing the scale-like ray and the dorsal marginal principal ray consecutively. The ventral margin of the caudal fin is formed by two unsegmented and two segmented ventral basal fulcra, and a series of fringing fulcra, which are laying on the second segmented basal fulcrum and the marginal principal ray.

## Discussion

### Taxonomy

Deecke [12] named the taxon *Archaeosemionotus connectens* on the basis the holotype only. De Alessandri [13] placed *A. connectens* [12] as a junior synonym of *Semionotus balsami* Bellotti [9]. According to the description of the holotype of *S. balsami* given by De Alessandri, the synonymy was probably right, but the holotype of *S. balsami* was lost in 1943 during World War II and, thus, it is currently impossible to compare the specimens and confirm the synonymy. For the same reason, and also considering that different fishes were simultaneously referred to *S. balsami* by De Alessandri [13], the nominal species *Semionotus balsami* should be regarded as a *nomen dubium*.

Following the synonymy of *Archaeosemionotus connectens* with *Semionotus balsami* indicated by De Alessandri [13] and based on the principle of priority, Bürgin et al. [26] proposed the new combination *Archaeosemionotus balsami* and referred to *Archaeosemionotus* sp. several new specimens of an actually different fish from the Prosanto Formation, which represents a new, still undescribed taxon. The main feature for the referral of the fish from the Prosanto Formation to *Archaeosemionotus* has been the presence of a mosaic of suborbital bones, which these authors understood as a distinctive feature of *A. balsami*. However, De Alessandri ([13]: p. 69) described only two large suborbitals in the cheek of the holotype of *S. balsami* and there are only two large and one small suborbital in the cheek of the holotype of *A. connectens*. This confusion is the consequence of the lost of the holotype of *S. balsami* on the one hand, and the simultaneous referral of different fishes to this species by De Alessandri [12] on the other. The mosaic of suborbitals is actually one of the main features distinguishing *A. connectens* from the fish of the Prosanto Formation. However, unaware of this difference and following Bürgin et al. [26], the name *Archaeosemionotus* stayed bound to the fish of the Prosanto Formation [32], [33], which has also been found in the Meride Limestone [34], [35], [36], [37]. Consequently, it is necessary to rectify the taxonomic status of *A. connectens* [12], which is here restricted to the holotype and the specimen PIMUZ A/I 552. The species of the Prosanto Formation and the Meride Limestone that has been referred to *Archaeosemionotus* represent a new neopterygian genus, which is currently under study (López-Arbarello and Stockar work in progress).

### Phylogenetic relationships

The presence of an interoperculum in *Archaeosemionotus connectens* indicates that the fish is a neopterygian. The combination of the large infraorbital bones forming the ventral margin of the orbit and the pattern of two large suborbitals resembles the skull of *Ophiopsis* [38] and *Furo* [19] very closely. According to Bartram [38], such a constellation of cheek and circumborbital bones is unique of Ophiopsidae among neopterygians and, thus, we explored the possible phylogenetic relationships of *A. connectens* with this family and closely related taxa.

### Discussion of characters

The complete list of characters and the data matrix are provided as (Text S1). Although most of the characters (characters 1–10, 13–24, and 26–53) and character scorings were taken directly from Grande and Bemis [15], some additions and modifications have been done after direct observation of specimens (detail information is available in Morphobank Project 1105 [20]). Characters 12 and 25 are modified from Grande and Bemis' characters 13, and 30 and 62, respectively.

Character 12: Urodermals in the caudal skeleton: present (0); absent (1); presence of a complete body lobe (2).

Grande and Bemis [15] did not distinguish character state 2, which represents a condition that they included in their character state 0 (presence of urodermals). Although the homology between the urodermals and rhomboid scales is widely accepted [39], [40] states 0 and 2 represent two clearly different conditions. In the first case, the urodermals are a few modified scales, with or without ganoine layer, which are placed lateral to the most dorsal principal caudal fin rays in fishes, the body of which is naked or covered with elasmoid scales. In the second case, there is a complete body lobe formed by several rows of rhomboid scales.

Character 25: Postmaxillary process: absent (0); present and small (1); present and thick and elongate (2).

Character states 0 and 1 coincide with character 62 of Grande and Bemis [15], i.e. the shape of the posterior margin of the maxilla. These authors defined a character state 0 for a margin convexly rounded or straight and a character state 1 for an excavated margin, which might be concave or has a posterior maxillary notch. When the posterior margin of the maxilla is convexly rounded or straight, a postmaxillary process is absent. Such a process is clearly present when a postmaxillary notch is present in the posterior margin of the maxilla, but it is also present when the margin is concave (e.g. *Solenhofenamia elongata*, *Ionoscopus cyprinoides*). In the tribe Vidalamiini, the postmaxillary process is uniquely enlarged and this condition is here represented with the character state 2, and in Grande and Bemis [15] with the character 30, which scores a postmaxillary process under postmaxillary notch tiny or absent (state 0) or thick and elongate (state 1).

Apart from characters 1–53, including the cases discussed above, other characters from Grande and Bemis [15] are not included here because they are uninformative for the present analysis.

Alvarado-Ortega and Espinoza-Arrubarrena [15] presented a cladistic analysis to explore the relationships of *Quetzalichthys perrilliatae*. Our characters 11, 54 to 57 are taken from this analysis. Among them, we modified the definition of two characters (their characters 15 and 6, respectively):

Character 55: Type of scales: rhomboid (0); of “amioid type” (1) (to apply the nomenclature of Schultze [41]).

Character 57: Vertebral centra: unossified (0); hemichordacentra, diplospondylous (1); solid perichordally ossified, diplospondylous (2); solid perichordally ossified, monospondylous (3).

### Cladistic analysis

**Figure 10:**
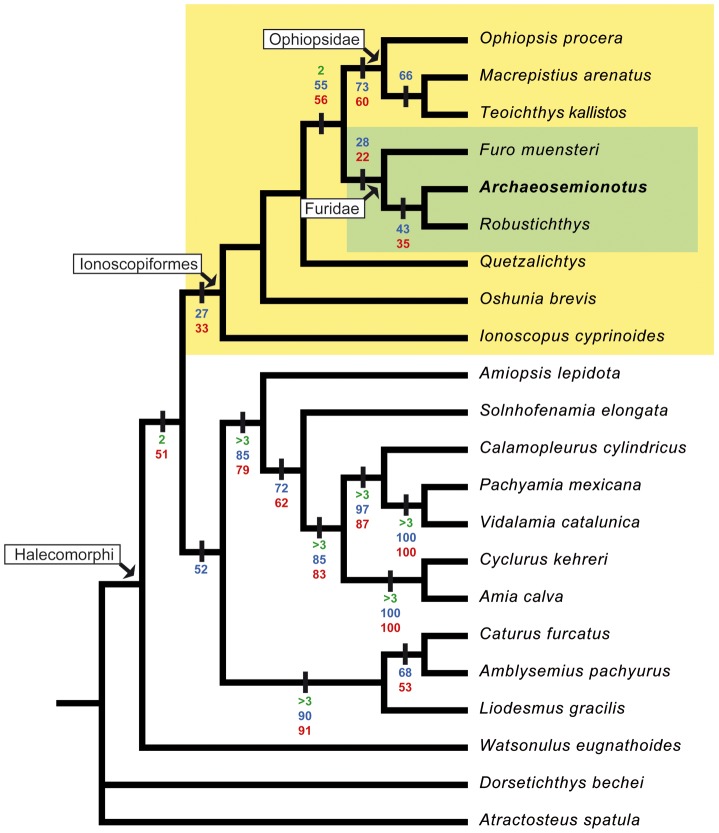
Phylogenetic relationships of *Archaeosemionotus connectens*. Single most parsimonious tree (Length = 124; CI = 0,5806; RI = 0,7306; HI = 0,4194; RC = 0,4242) obtained through branch and bound search with furthest addition sequence in PAUP* Version 4.0 beta version [21]. Bremer values higher than 1 and bootstrap and jackknife values higher than 50% (except for the nodes directly related to the relationships of *Archaeosemionotus*) are indicated on the branches with green, blue and red colours, respectively. [planned for 1,5-column width].

The cladistic analysis produced a single most parsimonious tree of 127 steps length (Fig. 10). *Archaeosemionotus connectens* is well nested within the Ionoscopiformes sensu Grande and Bemis [15], which is the sister-group of the Amiiformes. Within the ionoscopiform clade, *A. connectens* is the sister-taxon of *Robustichthys luopingensis* and they together form a monophyletic group with *Furo muensteri*. This clade constitutes the sister-group of the clade (*Ophiopsis procera* (*Macrepistius arenatus* + *Teoichthys kallistos*)) and we thus interpret them as the families Furidae and Ophiopsidae, respectively. These families are here understood as the most restrictive clades including *Furo* on the one hand and *Ophiopsis* on the other hand. These relationships are in agreement with previous phylogenetic analyses [15], [16], [42], although these studies do not include *Archaeosemionotus* or *Robustichthys*. In the cladistic analysis of Xu et al. [18], the relationships of *R. luopingensis* remain unresolved in a polytomy with a clade equivalent to our Ophiopsidae and a clade (*Quetzalichthys* + *Oschunia brevis* + *Ionoscopus*). The relationships within these two clades remain also unresolved [18]. Neither *Furo* nor *Archaeosemionotus* are included in the analysis of Xu et al. [18] and, thus, the incorporation of these two taxa in our analysis made the phylogenetic relationships of *Robustichthys* clear.

The complete list of apomorphies is included in the (Text S1) and only the nodes relevant to the relationships of *Archaeosemionotus* are discussed in this section. Four unambiguous synapomorphies support the sistergroup relationship between Furidae and Ophipsidae: smooth sided vertebral centra (ch. 3(2)); a short maxilla, which does not extend beyond the posterior margin of the orbit (ch. 47(1)); rhomboid scales (ch. 55(0)); and smooth surface of the lower circumborbital bones (ch. 56(0)). Among them, the second and fourth characters (47 and 56) are uniquely derived in this clade, but the latter is a reversal to the primitive condition in halecomorphs respect to the intensively pitted surface of the lower circumborbital bones present in basal ionoscopiforms (see below). The first of these characters (3), smooth sided vertebral centra, also derives once in the amiiforms above the level of *Amiopsis* (i.e. the Amiida sensu Grande and Bemis [15]). Character 55(1), the presence of rhomboid scales represents a reversal to the primitive condition in neopterygians.

The monophyly of Furidae is supported with two unambiguous synapomorphies. The first of these characters is the complete absence of sclerotic ossifications (ch. 13(1)), which otherwise occurs in *Cyclurus* and *Amia* among halecomorphs. Xu et al. [18] scored this feature as unknown (?) in *Robustichthys*, but we scored state 1 (absence) because neither the illustrated specimens nor the description show evidence for the possible presence of sclerotic bones in this fish. The second synapomorphy of Furidae concerns the shape of the anterior subinfraorbital bone in adult-sized individuals, which is short, subrectangular, and longer than deep (ch. 33(0)) in the fishes within this clade. However, this is the most generalized condition in neopterygians, and this synapomorphy represents a reversal from the apomorphic condition in ionoscopiforms, which is having a subrectangular and deeper than long anterior subinfraorbital (ch. 33(1)).

The number of supraorbital bones is very variable among halecomorphs, but the presence of more than four supraorbital bones (ch. 11(3)), usually arranged in more than one row, only occurs in *Calamopleurus*, *Furo* and within Ophiopsidae. There are only three to four supraorbitals in a single row in *Ophiopsis procera*. Therefore, this feature is synapomorphic of the ophiopsid clade only under accelerated transformation. Although two supraorbitals are preserved (Figs 5–6), it is not possible to be certain about their complete number in *Archaeosemionotus*. However, *Robustichthys* has two supraorbitals and this is the most probable condition in *Archaeosemionotus*.

The sister-group relationship between *Robustichthys* and *Archaeosemionotus* is supported with two unambiguous synapomorphies: strongly ornamented dermal skull bones (ch. 7(1)) and the absence of a postmaxillary process under the postmaxillary notch (ch. 25(0)). The first feature is homoplastic and also occurs in the derived ophiopsids (*Macrepistius arenatus* + *Teoichthys kallistos*) and in the amiid subfamily Amiinae (*Amia* and *Cyclurus*) within Halecomorphi. The second feature represents a reversal to the general condition in Neopterygii. Otherwise, the presence of a postmaxillary process under the postmaxillary notch (ch. 25(1)) is a synapomorphy uniquely derived in Halecomorphi [15].

The clade form by (*Ophiopsis procera* (*Macrepistius arenatus* + *Teoichthys kallistos*)) corresponds to the ophiopsid clade in the analysis of Alvarado-Ortega and Espinoza-Arrubarrena [16]. The clade is here supported with two unambiguous synapomorphies: dermopterotic and parietal bones of similar length (ch. 38(1)) and the presence of lateral line ossicles between the caudal fin rays (ch. 54(1)). The first of these characters is uniquely derived in this clade within Halecomorphi. The second feature is also known in *Amia calva* and *Calamopleurus cylindricus* [15] and since these ossicles or tubes are very delicate structures with very low preservation potential, this character might have a broader distribution than currently known.

In our analysis *Ionoscopus cyprinoides* is the most basal ionoscopiform and *Oshunia brevis* and *Quetzalichthys* are sequentially more closely related to the ophiopsids and furids than to *Ionoscopus* and the three taxa form the stem-group Ionoscopiformes. This pattern agrees with all previous cladistics analyses except Alvarado-Ortega and Espinoza-Arrubarrena [16] and Xu et al. [18]. In the first of these studies, *Quetzalichthys* and *Oshunia brevis* form a monophyletic group with *Ionoscopus*, representing the family Ionosocopidae, which in their analysis is the sister group of Ophiopsidae. According to Alvarado-Ortega and Espinoza-Arrubarrena [16] the clade containing (*Ionoscopus* (*Quetzalichthys* + *Oshunia*)) is supported by three characters. One of these alleged synapomorphies is the presence of two supraorbitals (our character 11, state 1), which is the condition present in *Quetzalichthys*. However, there are four supraorbital bones in *Ionoscopus cyprinoides* ([15]: fig. 410; [16]: Appendix), and the suborbitals are absent in *Oshunia* [15], [16]. Another synapomorphy of the clade (*Ionoscopus* (*Quetzalichthys* + *Oshunia*)) according to Alvarado-Ortega and Espinoza-Arrubarrena [16] is the presence of an intensively pitted ventral surface of the lower circumborbital bones (our character 56, state 1), but this is the plesiomorphic condition for ionoscopiforms in our analysis, which reverts in the clade (Ophiopsidae + Furidae).

The third character proposed by Alvarado-Ortega and Espinoza-Arrubarrena ([16]: 173) to support the monophyly of this clade is the “presence of well ossified vertebrae” referring to their character 6, which is based on Grande and Bemis' [15] character 1 and Gardiner et al.'s [43] characters 8 and 11. The definition of Alvarado-Ortega and Espinoza-Arrubarrena's character 6 is unclear. The vertebral centra of ionoscopiforms are solidly perichordally ossified, as is the case in amiiforms. The different conditions discussed by Grande & Bemis ([15]: 573) for their character 1 concern the presence or absence of diplospondylous perichordally ossified solid centra, which are absent in *Oshunia*, *Ionoscopus* and *Quetzalichthys* (the centra of which are monospondylous), and in several basal halecomorphs, which have diplospondylous centra, but they are not solidly ossified. Completing Alvarado-Ortega and Espinoza-Arrubarrena's character 6 and incorporating this information to our analysis (see character 57 above) did not produce a monophyletic Ionoscopidae including *Oshunia* and *Quetzalichthys*. According to the topology obtained in our analysis, the condition of the vertebral centra in *Ionoscopus*, *Oshunia* and *Quetzalichthys* is synapomorpic and uniquely derived in Ionoscopiformes, and the diplospondylous solid perichordally ossified centra is homoplastic, derived in the clade (*Ophiopsis procera* (*Macrepistius arenatus* + *Teoichthys kallistos*)) and in Amiidae independently.

The basal position of *Ionoscopus* and the Cretaceous *Quetzalichthys* and *Oshunia* imply very long ghost lineages, which indicate that the Ionoscopiformes diverged from its sister clade Amiiformes earlier than the Anisian (242–247 Ma) and, thus, the minimum estimate for this split might be taken within the Olenekian (247–251 Ma). However, although our data matrix does not support the monophyly of the Ionoscopidae sensu Alvarado-Ortega and Espinoza-Arrubarrena [16], making the grouping (*Ionoscopus* (*Quetzalichthys* + *Oshunia*)) monophyletic would take only 2 more steps and, thus, further research might revalidate this hypothesis. Under the hypothesis of the monophyly of the clade (*Ionoscopus* (*Quetzalichthys* + *Oshunia*)), the divergence date for the Ionosocpiformes might still be within the Anisian.

### The fossil record of ionoscopiforms

Previous to this study, reports of Triassic ophiopsids were dubious: *Ophiopsis* sp. in the Ladinian of Tarragona, Spain [44], *Ophiopsis* cf. *lariensis* and *Ophiopsis* cf. *lepturus* in the Ladinian of Switzerland [27], [34] and the record of *O*. *attenuata*, which is a Late Jurassic species, in the Norian of Austria [45]. The Spanish reports are based on very poorly preserved specimens, which have been referred to *Ophiopsis* based on their overall resemblance with the late Jurassic species of this genus, and the ionoscopiform relationships of these taxa have never been demonstrated on the basis of shared derived characters. Lombardo [3] excluded all the *Ophiopsis* cf. *lepturus* specimens studied by Bürgin [34] from the genus *Ophiopsis* and attributed them to a new genus and species *Daninia spinosa* within Perleidiformes. The same applies to the poorly preserved specimen referred to *Ophiopsis lariensis* De Alessandri [13] by Sieber [46], which comes from the Ladinian Partnachschichten of Weißenbach, Austria, and has been considered the oldest species of Ophiopsidae [16]. Furthermore, *Ophiopsis lariensis* [13] and other nominal species of putative ionoscopiforms from the Triassic are currently considered nomina dubia (e.g. *Pholidophorus ruppellii*, *Semionotus hermesii*; Table 1 in Table S1).

Other putative ionoscopiforms from the Triassic have been referred to the genus *Furo*, but it is doubtful whether these nominal species actually belong to this genus [47] and their taxonomic status needs revision. Lombardo [3] referred the specimen MCSNIO P456 (Civico Museo Insubrico di Storia Naturale di Induno Olona, Varese, Italy) from the Kalkschieferzone of the Meride Limestone to the species *Lepidotus trottii* Balsamo-Crivelli [8] and confirmed the referral of this species to the genus *Furo* ( = *Eugnathus*) proposed by De Alessandri [13]. However, the holotype of *L. trottii* is lost and the descriptions and illustrations of the type specimen do not show diagnostic features. Thus, we consider that *L. trottii* [8] is a *nomen dubium* and the taxonomic status of MCSNIO P456, the fish studied by Lombardo, should be revised. According to Lombardo [3] MCSNIO P456 not only has an overall morphology very similar to that of *A. connectens*, but it also presents exactly the same pattern of suborbital bones. The relative size and shape of the infraorbital bones forming the ventral margin of the orbit in MCSNIO P456 is however different than the condition in *A. connectens* and therefore, this specimen probably represents a second species of *Archaeosemionotus*.

**Figure 11:**
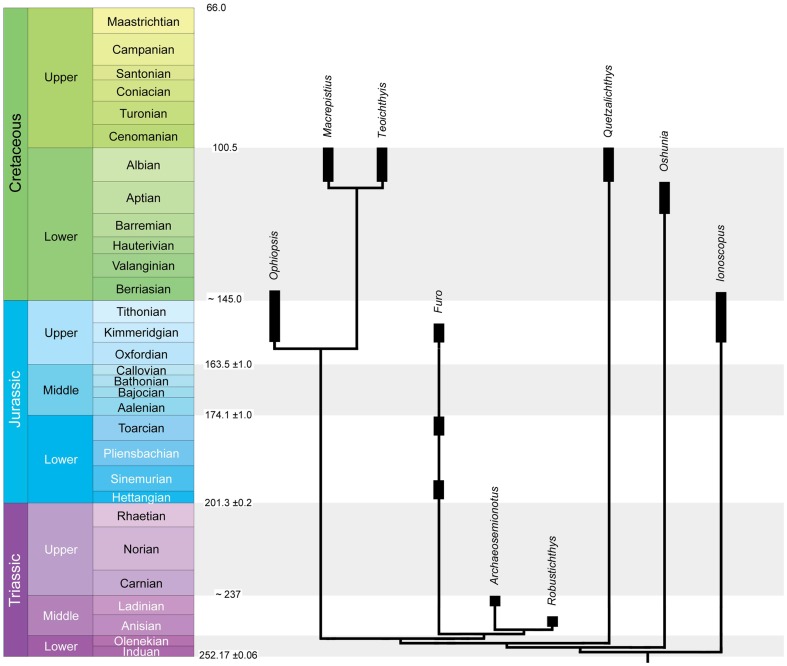
Figure 11 of PIMUZ A/I 552. Chronogram of Mesozoic ionoscopiforms based on the strict consensus tree shown in Figure 10. Stratigraphic range of the genera based on Xu et al. [18] for *Robustichthys*, Bartram [38] for *Ophiopsis*, Wenz [47] and Lane and Ebert [19] for *Furo*, Stützer [50] for *Ionoscopus*, and Alvarado-Ortega and Espinosa-Arrubarrena [16] and Machado et al. [53] for the Cretaceous taxa. Stratigraphic chart based on Cohen et al. [55]. [planned for 2-column width].

Although at least some of the Triassic species described above might represent basal ophiopsids or ionoscopiforms, they are in need of systematic revision and, thus, *Archaeosemionotus connectens* (Ladinian) and *Robustichthys luopingensis* (Ansian) [18] are currently the oldest and only confident records of Ionoscopiformes in the Triassic. The next oldest ionoscopiform genus is *Furo*, which is known from the Lower and Upper Jurassic. At least two Early Jurassic and one Late Jurassic species are valid: the type species *F. orthostomus* from the Hettangian-Sinemurian of Dorsetshire, England [48], *F. normandica* [47] from the Toarcian of Normandy, France, and *F. muensteri* from the Kimmeridgian of Bavaria, Germany ([19], [47], [49]; Fig. 11). Apart from the above-discussed doubtful records of *Ophiopsis* in the Triassic, this genus is represented with several species ranging from the Kimmeridgian (Late Jurassic) to the Berriasian (earliest Cretaceous) [37]. Although the genus *Ionoscopus* also needs taxonomic revision, according to Stützer [50] it is represented by species ranging from the Late Jurassic to the Early Cretaceous of Europe.

The remaining ionoscopiform genera have a more limited stratigraphic distribution. *Oshunia*, with a single species *O. brevis* [51] is only known in the Aptian of Chapada do Araripe, Brazil. *Quetzaichthys perrilliatae* [16] and *Teoichthys*, with two species *T. kallistos* [52] and *T. brevipina* [53] are so far only known from the Albian of the Tlayúa Quarry, Mexico. Finally, *Macrepistius arenatus* [54] is only known from the Albian Glen Rose Formation in Texas, USA.

Consequently, although patchy, the reliable fossil record of ionoscopiforms ranges from the Anisian (Middle Triassic) to the Albian (late Early Cretaceous) spanning approximately 140 Ma [55]. Therefore, these fishes constitute a long-living lineage within Mesozoic fish faunas and further studies on these fishes (in preparation by Lane [19] and Machado and collaborators [53] independently) will certainly help to elucidate the Mesozoic history of halecomorphs in particular and neopterygians in general. The results of our cladistic analysis show that further research on this group is needed because it shows enormous ghost lineages of at least 100 Ma (Fig. 11). Xu et al. [18] proposed a Palaeotethys east-west corridor dispersal hypothesis for the Ionoscopiformes. Such hypothesis is mainly based on tectonic evidence because no phylogenetic analysis of ionoscopiforms has shown any clear distributional pattern [16], [18]. Indeed, our cladogram does not directly support this idea because the two Triassic taxa are well nested within the most derived ionoscopiform clade whereas two of the Cretaceous taxa are part of the stem-group ionosocpiforms. Given the long stratigraphic range and patchy fossil record of the clade, it is to be expected that many more taxa can be referred to the Ionoscopiformes, both new taxa, and known taxa of currently uncertain relationships. It is obvious that recent efforts have concentrated on the later history of the group (Late Jurassic to Cretaceous), and more work is needed on their probably Triassic origin and early radiation in the Triassic and earlier stages of the Jurassic. Incorporating more early Mesozoic ionoscopiform taxa would fill the current ghost lineages and reveal a clear biogeographic pattern. Therefore, these studies are essential to elucidate the evolutionary history and to better evaluate the evolutionary and palaeoecological and palaeogeographic significance of the group.

## Supporting Information

Table S1 **Taxonomic status of the nominal species of the Perledo fauna and morphometric measurements.**
(DOCX)

Text S1 **Complete list of characters and data matrix for phylogenetic analysis.**
(DOCX)
