# Supplementary material for: Phylogenetic Relationships of the Triassic Archaeosemionotus Deecke (Halecomorphi, Ionoscopiformes) from the ‘Perledo Fauna’
Source: PLoS One. 2014 Oct 8;9(10):e108665. doi: 10.1371/journal.pone.0108665 (PMC4189917; doi:10.1371/journal.pone.0108665)
Supplement: Table S1 — Taxonomic status of the nominal species of the Perledo fauna and morphometric measurements. (DOCX) [file pone.0108665.s001.docx]

**Supporting Information Tables S1 to:**

**Phylogenetic relationships of the Triassic *Archaeosemionotus* Deecke (Halecomorphi, Ionoscopiformes) from the ‘Perledo fauna’**

Adriana López-Arbarello^1^*, Rudolf Stockar^2^ and Toni Bürgin^3^

^1^SNSB- Bavarian State Collection for Palaeontology and Geology, and GeoBio-Center Ludwig Maximilian University, Richard-Wagner-Strasse 10, D-80333 Munich, Germany, a.Lopez-Arbarello@lrz.uni-muenchen.de

^2^Museo Cantonale di Storia Naturale, Viale C. Cattaneo 4, CH-6900 Lugano, Switzerland, [rudolf.stockar@ti.ch](mailto:rudolf.stockar@ti.ch)

^3^Naturmuseum, Museumstrasse 32, CH-9000 St. Gallen, Switzerland, toni.buergin@naturmuseumsg.ch

**Table 1. Species of actinopterygians named by Bellotti [1] (left column) and their current taxonomic status (right column).**

| *Lepidotus serratus* | *Perleidus altolepis* [2] |
| --- | --- |
| *Pholidophorus oblongus* | *Perleidus altolepis* [2] |
| *Urolepis macropterus* | *Aneurolepis macroptera* [3] |
| *Urolepis microlepidotus* | *Aneurolepis macroptera* [3] |
| *Uroleis elongatus* | *Aneurolepis macroptera* [3,9] |
| *Semionotus bellotti* Rüppel | *Allolepidotus bellottii* [2,4] |
| *Semionotus inermis* | *Allolepidotus bellottii* [2,9] |
| *Pholidophorus porro* | *Prohalecites porroi* [8,9] |
| *Lepidotus pectoralis* | Nomen dubium [2,3] |
| *Semionotus brevis* | Nomen dubium [3] |
| *Semionotus balsami* | Nomen dubium [3,4] |
| *Semionotus dubius* | Nomen dubium [3,4,9] |
| *Pholidophorus ruppellii* | Nomen dubium [3] |
| *Pholidophorus lepturus* | Nomen dubium [3] |
| *Belonorhynchus robustus* Bellotti in [5] | *Saurichthys macrocephalus* [6] |
| *Semionotus hermesii* Bellotti in [5] | Nomen dubium [3] |

**Table 2. Actinopterygian taxa named by Deecke [7] (left column) and their current taxonomic status (right column).**

| *Semionotus altolepis* | *Perleidus altolepis* [2] |
| --- | --- |
| *Allolepidotus* | *Allolepidotus* [2] |
| *Allolepidotus nothosomoides* | *Allolepidotus bellottii* [2] |
| *Prohalecites* | *Prohalecites porroi* [8,9] |
| *Belonorhynchus macrocephalus* | *Saurichthys macrocephalus* [6] |
| *Archaeosemionotus connectens* | *Archaeosemionotus connectens* (this work) |

**Table 3. Species of actinopterygians named by De Alessandri [9] (left column) and their current taxonomic status (right column):**

| *Ophiopsis lariensis* | Nomen dubium [3] |
| --- | --- |
| *Heterolepidotus taramellii* | uncertain |
| *Heterolepidotus egidii venantii* | uncertain |
| *Heterolepidotus (?) bellottii* | *Ctenognathichthys bellottii* [10] |

**Table 4. Morphometric measurements (in mm) of the two specimens representing *Archaeosemionotus connectens* Deecke, 1886.**

Abbreviations: SL, standard length; HL, head length; OL, longitudinal diameter of the orbit; PreV, length from the tip of the snout to the origin of the pelvic fins; PreD, length from the tip of the snout to the origin of the dorsal fin; PreA, length from the tip of the snout to the origin of the anal fin.

| Specimen | SMF-P1238b | PIMUZ A/I 552 |
| --- | --- | --- |
| SL | 107 |  |
| HL | 29,5 | 24,5 |
| OL | 7,7 |  |
| PreV |  | 52,7 |
| PreD | 63,9 | 57,7 |
| PreA |  | 72 |

# References

1. Bellotti C (1857) Descrizione di alcune nuove specie di pesci fossili di Perledo e di altre località lombarde. In: Stoppani A, editor. Studii geologici e paleontologici sulla Lombardia. Milano: Biblioteca Politecnica. pp. 491-438.

2. Lombardo C (2001) Actinopterygians from the Middle Triassic of northern Italy and Canton Ticino (Switzerland): anatomical descriptions and nomenclatural problems. Rivista Italiana di Paleontologia e Stratigrafia 107: 345-369.

3. Tintori A, Lombardo C (1999) Late Ladinian fish faunas from Lombardy (North Italy): stratigraphy and paleobiology In: Arratia G, Schultze HP, editors. Mesozoic Fishes 2 – Systematics and Fossil Record. Munich: Verlag Dr. Friedrich Pfeil. pp. 495-504.

4. Tintori A, Lombardo C (2007) A new early Semionotidae (Semionotiformes, Actinopterygii) from the Upper Ladinian of the Monte San Giorgio area (Southern Switzerland and Northern Italy). Rivista Italiana di Paleontologia e Stratigrafia 113: 369-381.

5. Bassani F (1886) Sui fossili e sull'età degli scisti bituminosi triassici di Besano in Lombardia. Atti della Società Italiana di Scienze Naturali e del Museo Civico di Storia Naturale di Milano 29: 15-72.

6. Rieppel O (1985) Die Triasfauna der Tessiner Kalkalpen. XXV. Die Gattung *Saurichthys* (Pisces, Actinopterygii) aus der mittleren Trias des Monte San Giorgio, Kanton Tessin. Schweizerische Paläontologische Abhandlungen 108: 1-103.

7. Deecke W (1889) Über Fische aus verschiedenen Horizonten der Trias. Palaeontographica 35: 13-138.

8. Tintori A (1990) The actinopterygian fish *Prohalecites* from the Triassic of northern Italy. Palaeontology 33: 155-174.

9. De Alessandri G (1910) Studii sui Pesci Triasici della Lombardia. Memorie della Società Italiana di Scienze Naturali e Museo Civico di Storia Naturale di Milano 7: 1-145.

10. Tintori A (1998) *Ctenognathichthys bellottii* (De Alessandri, 1910): Nomenclatural problems and stratigraphical importance of this Middle Triassic actinopterygian fish. Rivista Italiana di Paleontologia e Stratigrafia 104: 417-422.
