# Supplementary material for: Phylogenetic Relationships of the Triassic Archaeosemionotus Deecke (Halecomorphi, Ionoscopiformes) from the ‘Perledo Fauna’
Source: PLoS One. 2014 Oct 8;9(10):e108665. doi: 10.1371/journal.pone.0108665 (PMC4189917; doi:10.1371/journal.pone.0108665)
Supplement: Text S1 — Complete list of characters and data matrix for phylogenetic analysis. (DOCX) [file pone.0108665.s002.docx]

**Supporting Information Text S1 to:**

**Phylogenetic relationships of the Triassic *Archaeosemionotus* Deecke (Halecomorphi, Ionoscopiformes) from the ‘Perledo fauna’**

Adriana López-Arbarello^1^*, Rudolf Stockar^2^ and Toni Bürgin^3^

^1^SNSB- Bavarian State Collection for Palaeontology and Geology, and GeoBio-Center Ludwig Maximilian University, Richard-Wagner-Strasse 10, D-80333 Munich, Germany, a.Lopez-Arbarello@lrz.uni-muenchen.de

^2^Museo Cantonale di Storia Naturale, Viale C. Cattaneo 4, CH-6900 Lugano, Switzerland, [rudolf.stockar@ti.ch](mailto:rudolf.stockar@ti.ch)

^3^Naturmuseum, Museumstrasse 32, CH-9000 St. Gallen, Switzerland, toni.buergin@naturmuseumsg.ch

### List of characters used in the cladistic analysis

1. Posterior extent of exoccipitals in adult-sized individuals (from [1]: character 2).

0 reaches posterior margin of occiput

1 does not reach posterior margin of occiput

2. Anteriorly projecting spine-like processes on neural and-or haemal arches (from [1]: character 3).

0 absent

1 present

3. Lateral fossae of vertebral centra of adult-sized individuals (from [1]: character 4).

0 present, with two pits on each side of most centra

1 present, with three or more pits on each side of most of the centra

2 absent, centra smoothsided

4. Number of supraneurals (from [1]: character 5).

0 15 or more

1 13 to 14

2 5 to 11

5. Articular ossification of lower jaw (from [1]: character 6).

0 a single element, or two elements tightly sutured to each other

1 two separate elements not in contact with each other

6. Presence/absence of suborbital bones (from [1]: character 7).

0 one or more present

1 absent

7. Strength of ornamentation on dermal bones of skull (from [1]: character 8).

0 weak and/or fine

1 strong, coarse

8. Hypural-ural centra fusion in adult-sized individuals (from [1]: character 9).

0 all hypurals autogenous (separate) from the ural centra

1 all but first hypural fused to corresponding centra

9. Presence/absence of large parapophyses fused to most of the abdominal centra (from [1]: character 10).

0 absent

1 present

10. Presence/absence of substantial scapulocoracoid ossification in adult-sized individuals (from [1]: character 11).

0 one or more elements present in the shoulder girdle

1 absent

11. Number of supraorbital bones (from [2]: character 22).

0 3-4

1 2

2 0

3 more than 4

12. Urodermals in the caudal skeleton (modified from [1]: character 13).

0 present

1 absent

2 presence of a complete body lobe

13. Presence/absence of sclerotic ring ossification (from [1]: character 14).

0 present

1 absent

14. Size and shape of dorsal fin (from [1]: character 15).

0 short, with straight to falcate margin, 14-25 segmented rays and 14- 25 proximal radials

1 medium long, with bow-shaped or straight margin, 30-34 segmented rays, and an estimated 30-34 proximal radials

2 very long, with bowshaped margin, 36-47 segmented rays, and 37-48 dorsal proximal radials

3 extremely long, with bow-shaped margin, 48-53 segmented rays, and 49-54 proximal radials]

15. Morphology of teeth on anterior coronoid and vomer (from [1]: character 16).

0 conical, with pointed tips

1 styliform, with broadly rounded or flattened tips

16. Parietal length (from [1]: character 18).

0 relatively long, with a width-to-length ratio not exceeding 0.90

1 relatively short, with a width-to-length ratio well exceeding 0.90

17. Number of ural centra (from [1]: character 19).

0 10 or fewer

1 11 to 22

18. Shape of preopercle (from [1]: character 20).

0 L-shaped

1 crescentshaped, long and narrow

2 crescent-shaped, wide in middle tapering dorsally and ventrally

3 ovoid

19. Morphology of caps of the jaw teeth in adult-sized individuals (from [1]: character 21).

0 round in cross-section, not sharply carinate

1 labiolingually compressed, sharply carinate (keeled)

20. Lateral edge of posttemporal in adult-sized individuals (from [1]: character 22).

0 shorter than length of anterior edge

1 elongate, about equal to or greater than width of anterior edge

21. Shape of posterior margin of caudal fin (from [1]: character 23).

0 forked

1 convexly rounded

2 straight and nearly vertical

22. Arrangement of vomerine teeth (from [1]: character 26).

0 tooth patch with two to several rows of teeth

1 tooth patch with only a single anterior marginal row, plus one or more teeth in a longitudinal series perpendicular to the anterior marginal row

23. Number of epurals (from [1]: character 28).

0 2-8

1 10-15

24. Shape of basipterygium (from [1]: character 29).

0 proximal end flat and widened anteriorly

1 proximal end long and rodlike, without significant widening anteriorly

25. Postmaxillary process under postmaxillary notch (from [1]: characters 30 and 62).

0 absent

1 present and small

2 present and thick and elongate

26. Morphology of pleural ribs (from [1]: character 31).

0 distal ends pointed or with rounded points

1 distal ends flatly truncated, even in large adults

27. Shape of gular (from [1]: character 32).

0 subtriangular or subrectangular with acute rounded anterior apex

1 broad, oval, without acute anterior apex

28. Peculiar ornamentation pattern of strongly defined, converging lines on opercles in adult-sized individuals (from [1]: character 33).

0 absent

1 present

29. Frontal width in adult-sized individuals (from [1]: character 34).

0 relatively wide, with a width-to-length ratio of 0.26 to -0.65

1 relatively narrow, with a width-to-length ratio of 0.13 to 0.21

30. Shape of dermopterotic (from [1]: character 35).

0 greatly widened posteriorly and tapered anteriorly

1 subrectangular, not substantially tapered anteriorly or widened posteriorly

31. Width of opercle (from [1]: character 36).

0 narrow, with width-toheight ratio of 0.56 to 1.06

1 wide, with width-toheight ratio in range of 1.07 to 1.39

32. Number of preural vertebral centra (preural centra = abdominal plus preural caudal centra) (from [1]: character 40).

0 40 to 73

1 75 to 82

33. Shape of anterior subinfraorbital bone in adultsized individuals (from [1]: character 43).

0 short, subrectangular, longer than deep

1 subrectangular, deeper than long

2 long, very thin, tubelike

3 posteriorly expansive, tapering anteriorly

34. Number of epaxial procurrent caudal fin rays (from [1]: character 44).

0 0 to 11

1 12 to 15

35. Presence/absence of fringing fulcra on median fins (from [1]: character 45).

0 present

1 absent

36. One-to-one arrangement of hypurals and caudal fin rays (from [1]: character 46).

0 last few hypurals each articulate with the bases of several caudal fin rays

1 each hypural normally bears a single caudal ray

37. Number of ossified ural neural arches (from [1]: character 47).

0 normally four or more

1 normally 2 or fewer

38. Dermopterotic length to parietal length (from [1]: character 50).

0 dermopterotic significantly longer

1 lengths about equivalent

39. Presence/absence of opisthotic (from [1]: character 51).

0 present

1 absent

40. Presence/absence of pterotic (from [1]: character 52).

0 present

1 absent

41. Shape of maxilla extremely slender and rod-like (from [1]: character 53).

0 no

1 yes

42. Number of branchiostegal rays (from [1]: character 54).

0 21 or fewer

1 22 or more

43. Numerous paired, block-like ural neural arch ossifications (from [1]: character 55).

0 absent

1 present

44. Dermosphenotic attachment to skull roof in adultsized individuals (from [1]: character 56).

0 loosely attached on the skull roof or hinged to the side of skull roof

1 firmly sutured into skull roof, forming part of it

45. Shape of rostral bone (from [1]: character 57).

0 plate-like or short tube-like, without lateral horns

1 roughly V-shaped, with lateral horns

46. Lacrimal shape (from [1]: character 58).

0 longer than deep, and smaller than orbit

1 deeper than long, and massive (about size of orbit)

47. Posterior extent of maxilla (from [1]: character 59).

0 extends to below posterior orbital margin

1 does not extend below posterior orbital margin

48. Presence/absence of lateral line canal in maxilla (from [1]: character 60).

0 absent

1 present

49. Symplectic involvement in jaw joint (from [1]: character 61).

0 does not articulate with lower jaw

1 distal end articulates with articular bone of lower jaw

50. Innerorbital flange of dermosphenotic (from [1]: character 63).

0 smooth, without sensory canal

1 = bearing sensory canal tube

51. Shape of haemal spines (from [1]: character 65).

0 spine-like or rodlike

1 broadly spatulate in the transverse plane

52. Relative size of uppermost postinfraorbital in adult-sized individuals (from [1]: character 66).

0 short, much shorter than lowermost postinfraorbital

1 long, about equal in length to lowermost postinfraorbital

53. Orientation of preural haemal and neural spines near caudal peduncle (from [1]: character 67).

0 positioned at about 250 to 450 from the horizontal

1 strongly inclined to nearly horizontal

54. Lateral line ossicles between caudal fin rays (from [2]: character 13).

0 absent

1 present

55. Type of scales (modified from [2]: character 15).

0 rhomboid

1 amioid

56. Ventral surface of lower circumborbital bones (from [2]: character 18).

0 smooth

1 intensely pitted

57. Vertebral centra (modified from [1,2])

0 unossified

1 hemichordacentra, diplospondylous

2 solid perichordally ossified, diplospondylous

3 solid perichordally ossified, monospondylous

### Data matrix used in the cladistic analysis

*Atractosteus spatula* 1000001010121000?0101100000100000101??0000000000000000

*Dorsetichthys bechei* 00?00000010001000?0?0?000000000000000000000000000010001

*Amiopsis lepidota* ?1100000000000000100100010000000001110??00011000100000102

*Solnhofenamia elongata* 112100000010000101001000100?0010211110?100011000100000102

*Calamopleurus cylindricus* 112111100030000012112100100000000011101100001000100001102

*Pachyamia mexicana* 112?1110001001001211101121111111?11110??000110001?0000102

*Vidalamia catalunica* ?12??1100010000012111011211111100011101?00011000100000102

*Cyclurus kehreri* 112211111121121101001000100000000011101100011000100100102

*Amia calva* 1?2211111121130001001000100000010011101100011000100101102

*Caturus furcatus* 00000000000000101100100100000003000001111111000101010101

*Amblysemius pachyurus* ?00?000001000010110000000000000000000??11111000101010101

*Liodesmus gracilis* ?00?0000?00000101100?0010000000?00010??11011000101010100

*Ionoscopus cyprinoides* 000000000000000?100010010000?000000000000011000110000113

*Ophiopsis procera* 0?21?000000200??01000??010?000001000?1??00011111?10001002

*Macrepistius arenatus* 002??01?0?32?1???1000?????000?00100??10000?1?111110001002

*Oshunia brevis* 000?00000020?00001000000?0000?001?00000100011001110000113

*Watsonulus eugnathoides* 0000000000200010300000?000000000000?00000000000100000000

*Furo muensteri* ??2??00?0?32100??10000??1?00?00?000??0??00?1?111???0?000?

*Archaeosemionotus* ?????01????2100??10?0???0?00000?000??0??0????11????0??00?

*Quetzalichtys perrilliatae* ?000?0001?12?01011000?0?1??00000100000??00011101?10000113

*Teoichthys* ??2??01???32?3?0?1?00??????0?00?100??1??00?11111???0?1002

*Robustichthys* ?????01???121000?1000???0?00010?000??0??00?1101111?0?000?

The following scorings for *Robustichthys* have been changed from Xu et al. [3]:

Character 13. Presence/absence of sclerotic bones. Xu et al. [3] leave a ?, but we scored it 1 (absent) because there is absolutely no evidence for sclerotic bones in the specimens they figured. They also do not discuss this feature, so we can only base on their illustrations.

Character 18. Shape of preopercle. It is state 1 and not 2. Character state 2 refers to the condition in *Calamopleurus*, *Vidalamia* and *Pachyamia*, in which the preopercle tappers anteroventrally, but the preopercle of *Robustichthys* is similar to the preopercle of *Oshunia* and other ionosocpiforms (see Grande & Bemis [1]: character 20 and figure 242).

Character 27. Shape of gular. Similar case as character 18. Xu et al. [3] scored state 1, which is a peculiarly oval gular present in *Pachyamia* and *Vidalamia*. The gular of *Robustichthys* is visible in their figure 1c and has a straight to slightly concave lateral border. Therefore, it is rather subrectgangular as in most halecomorphs and so we scored state 0 (see Grande and Bemis [1]: character 32 and figure 136).

Character 34. Number of epaxial procurrent caudal fin rays. In the case of *Robustichthys* this character refers to the number of dorsal paired basal fulcra (see Grande and Bemis [1]: character 44), which are 11 in the photograph of the caudal peduncle in Xu et al.'s figure S1. Thus, we scored state 0.

# References

1. Grande L, Bemis WE (1998) A comprehensive phylogenetic study of amiid fishes (Amiidae) based on comparative skeletal anatomy. An empirical search for interconnected patterns of natural history. Society of Vertebrate Paleontology Memoir 4: 1-690; supplement to Journal of Vertebrate Paleontology 18.

2. Alvarado-Ortega J, Espinosa-Arrubarrena L (2008) A New genus of ionoscopiform fish (Halecomorphi) from the Lower Cretaceous (Albian) lithographic limestones of the Tlayúa Quarry, Puebla, Mexico. Journal of Paleontology 82(1): 163-175.

3. Xu G-H, Zhao L-J, Coates MI (2014). The oldest ionoscopiform from China sheds new light on the early evolution of halecomorph fishes. Biology Letters 10: 20140204.

### List of apomorphies


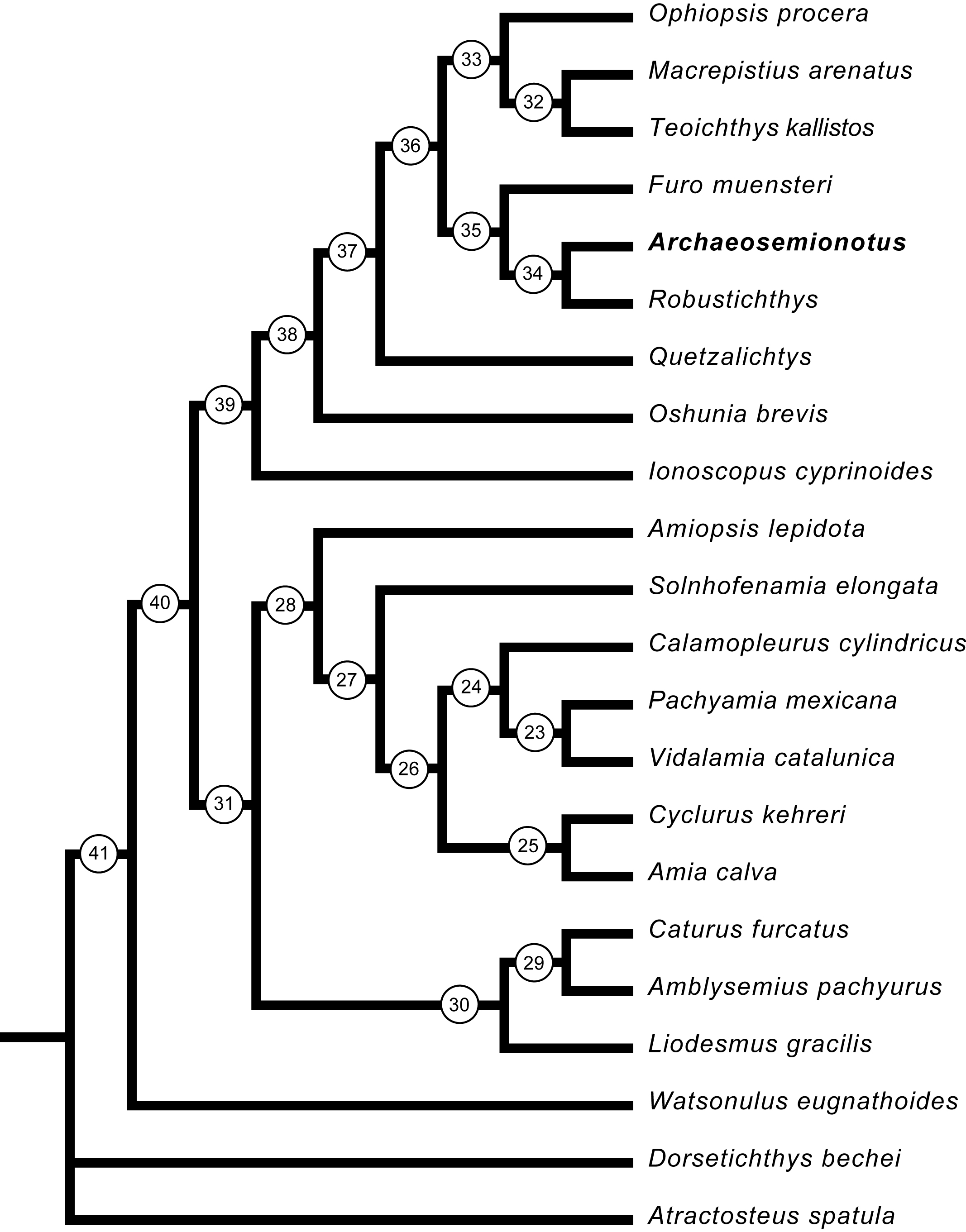


| Branch | Character | CI | Change | ACCTRAN | DELTRAN |
| --- | --- | --- | --- | --- | --- |
| Root --> node_41 | 11 | 0.300 | 1==>0 |  |  |
|  | 18 | 1.000 |  | 0-->1 |  |
|  | 49 | 1.000 | 0==>1 |  |  |
| node_41 --> node_40 | 18 | 1.000 |  |  | 0-->1 |
|  | 25 | 0.500 | 0==>1 |  |  |
|  | 44 | 0.500 | 0==>1 |  |  |
|  | 45 | 1.000 | 0==>1 |  |  |
|  | 55 | 0.500 | 0==>1 |  |  |
| node_40 --> node_31 | 3 | 0.667 |  | 0-->1 |  |
|  | 37 | 0.500 |  | 0-->1 |  |
|  | 39 | 1.000 | 0==>1 |  |  |
|  | 40 | 0.500 | 0==>1 |  |  |
| node_31 --> node_28 | 1 | 0.333 |  | 0-->1 |  |
|  | 2 | 1.000 | 0==>1 |  |  |
|  | 21 | 0.667 | 0==>1 |  |  |
|  | 35 | 1.000 | 0==>1 |  |  |
|  | 36 | 0.500 | 0==>1 |  |  |
|  | 37 | 0.500 |  |  | 0-->1 |
|  | 57 | 0.600 | 0==>2 |  |  |
| node_28 -->*Amiopsis lepidota* | 3 | 0.667 |  |  | 0-->1 |
| node_28 --> node_27 | 1 | 0.333 |  |  | 0-->1 |
|  | 3 | 0.667 |  | 1-->2 | 0-->2 |
|  | 4 | 0.667 | 0==>1 |  |  |
|  | 11 | 0.333 | 0==>1 |  |  |
| node_27 --> *Solnhofenamia elongata* | 16 | 0.200 | 0==>1 |  |  |
|  | 31 | 0.500 | 0==>1 |  |  |
|  | 33 | 0.750 | 0==>2 |  |  |
|  | 34 | 0.500 | 0==>1 |  |  |
| node_27 --> node_26 | 5 | 1.000 | 0==>1 |  |  |
|  | 6 | 1.000 | 0==>1 |  |  |
|  | 7 | 0.250 | 0==>1 |  |  |
| node_26 --> node_24 | 17 | 0.500 | 0==>1 |  |  |
|  | 18 | 1.000 | 1==>2 |  |  |
|  | 19 | 0.333 | 0==>1 |  |  |
|  | 20 | 1.000 | 0==>1 |  |  |
| node_24 --> *Calamopleurus cylindricus* | 11 | 0.300 | 1==>3 |  |  |
|  | 21 | 0.667 | 1==>2 |  |  |
|  | 22 | 0.250 | 0==>1 |  |  |
|  | 44 | 0.500 | 1==>0 |  |  |
|  | 54 | 0.333 | 0==>1 |  |  |
| node_24 --> node_23 | 23 | 1.000 | 0==>1 |  |  |
|  | 24 | 1.000 | 0==>1 |  |  |
|  | 25 | 0.500 | 1==>2 |  |  |
|  | 26 | 1.000 | 0==>1 |  |  |
|  | 27 | 1.000 | 0==>1 |  |  |
|  | 28 | 1.000 | 0==>1 |  |  |
|  | 29 | 1.000 | 0==>1 |  |  |
|  | 30 | 0.333 | 0==>1 |  |  |
|  | 31 | 0.500 | 0==>1 |  |  |
| Branch | Character | CI | Change | ACCTRAN | DELTRAN |
| node_23 --> *Pachyamia mexicana* | 14 | 0.600 | 0==>1 |  |  |
|  | 32 | 0.500 | 0==>1 |  |  |
|  | 34 | 0.500 | 0==>1 |  |  |
| node_26 --> node_25 | 4 | 0.667 | 1==>2 |  |  |
|  | 8 | 1.000 | 0==>1 |  |  |
|  | 9 | 0.333 | 0==>1 |  |  |
|  | 10 | 1.000 | 0==>1 |  |  |
|  | 11 | 0.300 | 1==>2 |  |  |
|  | 12 | 0.500 | 0==>1 |  |  |
|  | 13 | 0.333 | 0==>1 |  |  |
|  | 14 | 0.600 |  | 0-->2 |  |
|  | 52 | 1.000 | 0==>1 |  |  |
| node_25 --> *Cyclurus kehreri* | 14 | 0.600 |  |  | 0-->2 |
|  | 15 | 0.500 | 0==>1 |  |  |
|  | 16 | 0.200 | 0==>1 |  |  |
| node_25 --> *Amia calva* | 14 | 0.600 |  | 2-->3 | 0-->3 |
|  | 32 | 0.500 | 0==>1 |  |  |
|  | 54 | 0.333 | 0==>1 |  |  |
| node_31 --> node_30 | 16 | 0.200 | 0==>1 |  |  |
|  | 19 | 0.333 | 0==>1 |  |  |
|  | 41 | 1.000 | 0==>1 |  |  |
|  | 42 | 1.000 | 0==>1 |  |  |
|  | 51 | 1.000 | 0==>1 |  |  |
|  | 53 | 0.500 | 0==>1 |  |  |
| node_30 --> node_29 | 37 | 0.500 |  | 1-->0 |  |
|  | 43 | 1.000 | 0==>1 |  |  |
|  | 57 | 0.600 | 0==>1 |  |  |
| node_29 --> *Caturus furcatus* | 22 | 0.250 | 0==>1 |  |  |
|  | 33 | 0.750 | 0==>3 |  |  |
| node_29 --> *Amblysemius pachyurus* | 11 | 0.300 | 0==>1 |  |  |
|  | 25 | 0.500 | 1==>0 |  |  |
| node_30 -->*Liodesmus gracilis* | 37 | 0.500 |  |  | 0-->1 |
| node_40 --> node_39 | 50 | 1.000 | 0==>1 |  |  |
|  | 56 | 0.500 | 0==>1 |  |  |
|  | 57 | 0.600 | 0==>3 |  |  |
| node_39 --> *Ionoscopus cyprinoides* | 1 | 0.333 | 0==>1 |  |  |
|  | 22 | 0.250 | 0==>1 |  |  |
| node_39 --> node_38 | 11 | 0.300 |  | 0-->1 |  |
|  | 33 | 0.750 | 0==>1 |  |  |
|  | 48 | 1.000 | 0==>1 |  |  |
| node_38 --> node_37 | 12 | 0.500 | 0==>2 |  |  |
|  | 46 | 0.500 | 0==>1 |  |  |

| Branch | Character | CI | Change | ACCTRAN | DELTRAN |
| --- | --- | --- | --- | --- | --- |
| node_37 --> node_36 | 3 | 0.667 | 0==>2 |  |  |
|  | 4 | 0.667 |  | 0-->1 |  |
|  | 11 | 0.300 |  | 1-->3 |  |
|  | 47 | 1.000 | 0==>1 |  |  |
|  | 55 | 0.500 | 1==>0 |  |  |
|  | 56 | 0.500 | 1==>0 |  |  |
|  | 57 | 0.600 |  | 3-->2 |  |
| node_36 --> node_33 | 38 | 0.500 | 0==>1 |  |  |
|  | 54 | 0.333 | 0==>1 |  |  |
|  | 57 | 0.600 |  |  | 3-->2 |
| node_33 --> *Ophiopsis procera* | 4 | 0.667 |  |  | 0-->1 |
|  | 11 | 0.300 |  | 3-->0 |  |
| node_33 --> node_32 | 7 | 0.250 | 0==>1 |  |  |
|  | 11 | 0.300 |  |  | 0-->3 |
|  | 14 | 0.600 |  | 0-->1 |  |
| node_32 --> *Macrepistius* | 14 | 0.600 |  |  | 0-->1 |
| node_32 --> *Teoichthys* | 14 | 0.600 |  | 1-->3 | 0-->3 |
| node_36 --> node_35 | 13 | 0.333 | 0==>1 |  |  |
|  | 33 | 0.750 | 1==>0 |  |  |
| node_35 --> *Furo muensteri* | 11 | 0.300 |  |  | 0-->3 |
| node_35 --> node_34 | 7 | 0.250 | 0==>1 |  |  |
|  | 11 | 0.300 |  | 3-->1 |  |
|  | 25 | 0.500 | 1==>0 |  |  |
| node_34 --> *Robustichthys* | 11 | 0.300 |  |  | 0-->1 |
|  | 30 | 0.333 | 0==>1 |  |  |
|  | 46 | 0.500 | 1==>0 |  |  |
| node_37 --> *Quetzalichtys*  *perrilliatae* | 9 | 0.333 | 0==>1 |  |  |
|  | 11 | 0.300 |  |  | 0-->1 |
|  | 15 | 0.500 | 0==>1 |  |  |
|  | 17 | 0.500 | 0==>1 |  |  |
| node_38 --> *Oshunia brevis* | 11 | 0.300 |  | 1-->2 | 0-->2 |
|  | 40 | 0.500 | 0==>1 |  |  |
| node_41 --> *Watsonulus eugnatoides* | 12 | 0.500 | 0==>2 |  |  |
|  | 16 | 0.200 | 0==>1 |  |  |
|  | 18 | 1.000 |  | 1-->3 | 0-->3 |
